# Monitoring the VDPV2 outbreak in Egypt during 2020–2021 highlights the crucial role of environmental surveillance and boosting immunization in combating Poliovirus

**DOI:** 10.1186/s12879-024-09731-0

**Published:** 2024-08-26

**Authors:** Menna R. Shabana, Amira Y. Zaghloul, Tamer H. El Shaarawy, Nooran S. Elleboudy, Khaled M. Aboshanab

**Affiliations:** 1 WHO Regional Reference lab for Diagnosis of Poliovirus and Enteroviruses, VACSERA Dokki, Giza, Cairo, Egypt; 2 Department of Microbiology and Immunology, Faculty of Pharmacy, Organization of African Union, Ain Shams University, St. Abbassia, Cairo, 11566 Egypt

**Keywords:** Vaccine-derived polioviruses (VDPVs), Oral poliovirus vaccine, Environmental surveillance, World Health Organization, Sewage, Stool

## Abstract

**Background:**

Poliovirus is a highly infectious enterovirus (EV) that primarily affects children and can lead to lifelong paralysis or even death. Vaccine-derived polioviruses (VDPVs) are a great threat since they are derived from the attenuated virus in the Oral Poliovirus Vaccine (OPV) and can mutate to a more virulent form. The purpose of this study was to identify VDPV serotype 2 through the year 2020–2021 via surveillance of sewage samples collected from different localities and governorates in Egypt and stool specimens from Acute Flaccid Paralysis (AFP) cases. Both were collected through the national poliovirus surveillance system and according to the guidelines recommended by the WHO.

**Methods:**

A total of 1266 sewage samples and 3241 stool samples from January 2020 to December 2021 were investigated in the lab according to World Health Organization (WHO) protocol for the presence of Polioviruses by cell culture, molecular identification of positive isolates on L20B cell line was carried out using real-time polymerase chain reactions (RT-PCR). Any positive isolates for Poliovirus type 2 and isolates suspected of Vaccine Derived Poliovirus Type 1 and type 3 screened by (VDPV1) or Vaccine Poliovirus Type 3 (VDPV3) assay in RT-PCR were referred for VP1 genetic sequencing.

**Results:**

The outbreak was caused by circulating VDPV2 (cVDPV2) strains started in January 2021. By the end of February 2021, a total of 11 cVDPV2s were detected in sewage samples from six governorates confirming the outbreak situation. One additional cVDPV2 was detected later in the sewage sample from Qena (June 2021). The first and only re-emergence of VDPV2 in stool samples during the outbreak was in contact with Luxor in June 2021. By November 2021, a total of 80 VDPVs were detected. The Egyptian Ministry of Health and Population (MOHP), in collaboration with the WHO, responded quickly by launching two massive vaccination campaigns targeting children under the age of five. Additionally, surveillance systems were strengthened to detect new cases and prevent further spread of the virus.

**Conclusion:**

The continued threat of poliovirus and VDPVs requires ongoing efforts to prevent their emergence and spread. Strategies such as improving immunization coverage, using genetically stable vaccines, and establishing surveillance systems are critical to achieving global eradication of poliovirus and efficient monitoring of VDPVs outbreaks.

**Supplementary Information:**

The online version contains supplementary material available at 10.1186/s12879-024-09731-0.

## Background

Polioviruses (PVs) are the most notorious enteroviruses (EVs) being the causative agent of paralysis in children leading to life-long disability [1]. Three serotypes are identified for Polioviruses: PV1, PV2, and PV3, all causing poliomyelitis in humans [2].

Although OPV has multiple strengths, limitations do exist [3]. The two main drawbacks are the development of vaccine-associated paralytic poliomyelitis (VAPP) in vaccinated cases as well as those who contact them, and the development of genotypically different VDPVs, especially in immunocompromised individuals or during epidemics [4]. The VDPVs have received increased attention as they cause AFP with comparable severity to the wild-type polioviruses; hence their emergence affects the future of the polio endgame [5, 6]. The categorization of polioviruses is done through sequencing of the major capsid protein-encoding region VP1 comprising 12% of the genome (around 900 nucleotides) [7].

A poliovirus, serotypes (PV1, PV2, or PV3), may be either a wild-type virus or a vaccine-related one. Vaccine-related viruses are further categorized into Sabin-like polioviruses (SLPVs) with minimal variation from vaccine strains and VDPVs where the variation in VP1 sequence is more than 1% for PV1 and PV3 and 0.6% for PV2. The World Health Organization (WHO) categorizes VDPVs into three groups: circulating VDPVs (cVDPVs), immunodeficiency-associated VDPVs (iVDPVs), and ambiguous VDPVs (aVDPVs). cVDPVs are identified when there is clear evidence of transmission within a community. iVDPVs are found in individuals with weakened immune systems. aVDPVs are isolated from individuals without immunodeficiency or from wastewater samples, and their classification is uncertain due to the lack of evidence for community transmission [8].

After eradication of wild-type PV2 & withdrawal of its serotype from the OPV in 2016, detections of VDPV2 in clinical or environmental samples is considered of special importance to the polio eradication program. Regarding Egypt, the last detection of VDPV2 before switching to the bivalent OPV was in April 2016 in a sewage sample collected from North Sinai. After switching to the bOPV in May 2016, no VDPV2 was detected in sewage or in stool samples from AFP cases until 2020. The WHO has reported that from the beginning of 2017, various cVDPV2 outbreaks caused by strains of different genotypes occurred in Africa which has led the WHO to respond using outbreak plans in 21 countries in central Africa [9]. This may highlight the importance of keeping a strong and efficient poliovirus surveillance system in all countries, especially, those in high & moderate risk areas.

Accordingly, this study was aimed at monitoring VDPV2 via surveillance of sewage samples collected from different localities and governorates in Egypt through the year 2020/2021 as well as stool specimens collected from AFP cases whenever available. The surveillance methods used for the detection and identification of the isolated poliovirus were done according to the standard protocols and guidelines of the WHO.

## Materials and methods

### Study design

The sewage samples were collected monthly (from January 2020 to December 2021) from the same site of sewage treatment plants all over the 27 Egyptian governorates for surveillance purposes in Poliovirus detections. The collection of samples (≈ 1 L each) was done by the Grab sampling method according to the GPEI guidelines [8].

### Collection of stool specimens

Two stool specimens (24 h apart) from AFP cases selected according to the criteria recommended in WHO guidelines and were collected from all 27 Egyptian governorates. This study adhered to the Declaration of Helsinki and followed all relevant local and national regulations and was approved from the Ethics Committee Faculty of Pharmacy at Ain Shams University, Cairo, Egypt (ACUC-FP-ASU RHDIRB2020110301 REC # 30). A written informed consent was taken from the suspected cases upon clarifying the purpose of the study.

### Processing of sewage samples

Processing of sewage samples (*n* = 1266) were collected from January 2020 to December 2021 from all 27 Egyptian governorates was performed immediately after admission. The two-phase concentration technique was used as previously reported [8]. Briefly, 500 mL of sample were processed by centrifugation at 1500 xg for 20 min at 4 °C. The clear supernatant was collected and homogenized with 39.5 mL of 22% Dextran solution (Serva, Germany); 287 mL of 29% PEG 6000 solution (Sigma-Aldrich, Switzerland); and 35 mL 5 N NaCl (Sigma-Aldrich, Switzerland) for 60 min in ice bath using a magnetic stirrer. To separate the polymers, the homogenate was left to stand in a separation funnel overnight at 4 °C and the lower phase with the interphase were collected by dripping. The pellet (collected initially by sample centrifugation) was left to dry overnight then re-suspended in the concentrated supernatant before shaking with chloroform (Acros, Belgium) for 20 min in shaker at maximum speed. Lastly, centrifugation was done at 2000 ×g for 20 min at 4 °C prior to virus detection. The concentrates were stored at − 20 °C for further use [10].

### Processing of stool samples

This was done as specified by the WHO protocol [10]. In brief, 2 g of the stool specimen was mixed with PBS (10 mL), 1% chloroform (1 mL), 5 glass beads and shaken vigorously for 20 min then centrifuged at 1500 xg for 20 min at 4 °C. Supernatants were collected and stored at -20 °C.

### Cell culture

Human Rhabdomyosarcoma (RD cells, ATCC cat# CCL-136) and L20B cells (mouse L cells transfected with the gene for the human cellular receptor for poliovirus) were obtained from Center for Disease Control and Prevention (CDC), USA [10], and were used to isolate enteroviruses and poliovirus respectively. Both cell lines were grown using minimum essential medium (MEM) with Earle’s balanced salts and fetal bovine serum (FBS) (Sigma Aldrich, USA). The cell culture to inoculate sewage concentrates was performed using T25 plastic cell culture flasks and cell culture tubes used to inoculate stool suspension using Minimum Essential Medium (MEM) supplemented with 10% fetal bovine serum (FBS). For cell maintenance, MEM supplemented with 2% FBS was utilized. Incubation of pre-prepared cell lines for inoculation was carried out at a temperature of 37 °C for duration of 48 h [10].

### Inoculation of sewage samples

For each sample, the extracted concentrate was used to inoculate five 25 cm^2^ flasks containing fresh monolayer cultures of L20B (E1, E2, E3, E4, E5) and 1 flask containing RD fresh cultures (0.5 mL to each flask). Incubation was done at 37 °C and flasks were examined daily for cytopathic effect (CPE) for 5 days. In cases where the initial set of samples did not exhibit CPE, they were subsequently re-passaged onto a new set of flasks and observed them. If no CPE was detected after daily screening for a period of 10 days, the samples were considered negative and subsequently discarded [11].

If the CPE were observed on L20B cells, the corresponding samples underwent two cycles of freezing and thawing before being utilized for re-passage on RD cell line. Polymerase Chain Reaction (PCR) analysis was employed to confirm the specific type of poliovirus present in the positive samples.

### Inoculation of stool samples

Monolayers (90% confluence) of RD and L20B cells in tube were inoculated with 0.2 mL of each of the stool suspension supernatants where each sample has its own lab serial number, incubated at 37 °C, and visualized via inverted microscope on daily basis for 5 days. Re-passaged samples were also tested over a 5-day period, before disposal. Samples showing CPE were considered positive and were kept at -20 °C for PCR testing [11].

### Real time -polymerase chain reaction (rRT-PCR)

The rRT-PCR is a tool used to detect the type of PV presented in the samples, it was done by following a specific algorithm to differentiate between different types of isolates. The rRT-PCR screening kit, ITD 5.1, consists of six assays and reaction conditions were used as previously reported [10]. The target genes and the list of primers is displayed in Tables S1 and S2.

Each ITD PCR reaction consisted of 10 µL of qScript™ XLT One-Step RT-qPCR ToughMix^®^ (Quanta Biosciences, Beverly, MA, USA), 1 µL of primers/probe(s) mix (contained in the ITD kit; CDC, Atlanta, GA, USA), 8 µL RNase-free water, and 1 µL of template (virus culture supernatant or RNA). The RT–PCR was done by incubating 1 µL sample with 19 µL of Master Mix, using 96 well Applied Biosystem 7500 Real Time PCR System (ABI7500, ThermoFisher Scientific, Waltham, MA, USA) that depends on TaqMan Assay. Positive results were considered by comparing the Ct-values of the samples against the negative control as per instructions in the kit pamphlet [10].

### Sequencing of poliovirus isolates

Genetic sequencing is the definitive method for confirming vaccine-derived polioviruses (VDPVs) by comparing their sequences with Sabin prototypes, thereby identifying the presence of mutations. When Poliovirus Type 2 virus and Poliovirus isolates suspected of being VDPVs showed negative results for the VP1 gene in the VDPV assay using real-time RT-PCR, they were subjected to sequencing for further analysis [13].

### RNA extraction

About 140 µL of culture isolate were used for RNA extraction by QIAamp viral RNA extraction kit (QIAgen, Cat# 52904) according to the manufacturer’s instructions. The purified RNA was eluted in 50 µL of AVE buffer and concentration was measured by Nanophotometer N60 (Implen Nanophotometer) [10].

### Amplification of VP1 gene by RT-PCR

The purified RNA was used as template to amplify the region containing the VP1 gene using Y7R & Q8 as forward & reverse primers respectively and QIAgen One-step RT-PCR kit (QIAgen, Cat#151019502) according to the following protocol. For each 50 µL reaction mixture the following were added: 10 µL of 5x reaction buffer, 2 µL of dNTPs (10 mM each, Roche#11814362001), 2 µL of enzyme mix, 0.25 µL of protector RNAse inhibitor (Roche#3335402001), 1 µL of each primer (40 pmole/µL for Y7R & 10 pmole/µL for Q8), 3–5 µL of sample (100–250 ng of template RNA) and sufficient quantity of nuclease free water (Thermo Fisher Scientific, Waltham, MA, USA CAT# AM9937 ) to a final volume of 50 µL. ProFlex 96-Well PCR System (Thermofisher, Waltham, MA, USA, CAT#4375305 ) was programmed to the following thermal profile: 50 °C for 30 min, 95 °C for 15 min, then 35 cycles of 94 °C for 30 s, 42 °C for 45 s and 60 °C for 1 min, then one stage of post-extension at 60 °C for 5 min [14].

The amplification product of RT-PCR (about 1100 bp) was visualized on 1% agarose gel (GeneMate Gene Pure LE Agarose, BioExpress #E-120-500) with pre-added Ethidium Bromide (0.5 µg/mL) in comparison to 100 bp DNA Ladder (Invitrogen# 15628050). The amplicon was directly purified from the reaction using QIAquick PCR purification Kit (QIAgen Cat#. 28104 and 28106). The purified DNA amplicon was eluted in 50 µL of NF water and its concentration was measured by Implen NanoPhotometer^®^ N60 (FisherScientific Co., Denmark) [14].

### Cycle sequencing of the purified VP1 gene

About 20–40 ng (usually 0.5–1 µL) of the purified amplicon was used as template for the cycle sequencing reaction using BigDye Terminator Cycle Sequencing Kit (ThermoFisher Scientific, Waltham, MA, USA, Cat#4458688) according to the following protocol: for each 10 µL reaction mixture the following were added: 2 µL of BigDye sequencing enzyme mix, 1 µL of 5x BigDye sequencing buffer, 0.5 µL of each primer and sufficient quantity of nuclease free water to a final volume of 10 µL. Proflex thermal cycler was programmed to the following thermal profile: 25 cycles of 94 °C for 20 s, 42 °C for 15 s and 60 °C for 4 m’, then hold at 4 °C [13].

### Cycle sequencing primers

A set of 4–8 primers (Supplied by Polio and Picornavirus Laboratory Branch, CDC, Atlanta, GA USA) was used to sequence the VP1 gene; each set was composed of a mixture of both generic & type-specific primers in both sense & antisense directions, as shown in Table S2 [13]. The product of the cycle sequencing reaction was purified using AxyPrep MagDyeClean (Axygen, (Ca, USA Cat#94587) as per manufacturer’s instructions & loaded to ABI3500 Genetic Analyzer (ThermoFisher Scientific, Waltham, MA, USA).

### Sequence analysis

The resulting sequence fragments were assembled, edited & analyzed using Sequencher 5.4.1 software (GeneCodes Corporation, Ann Arbor, USA) to obtain the complete VP1 sequence for each sample. The obtained consensus sequence is compared to the VP1 sequence of the Sabin reference strain for each serotype to differentiate Sabin from vaccine-derived polioviruses. Consensus sequences of VDPVs were also shared with CDC for comparison with the global polio sequence database & identifying the genetic relations of the isolated VDPVs [13].

## Results

### Detection of polioviruses from sewage samples

**Fig. 1:**
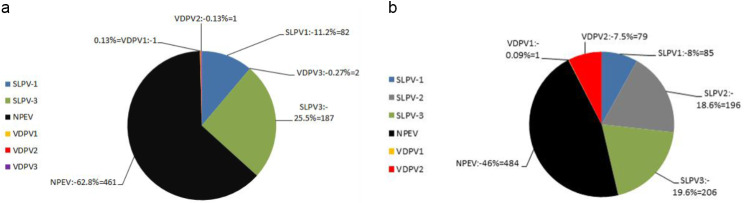
Distribution of detected viral isolates from sewage samples in the years 2020 (**a**) and 2021 (**b**). SLPV-1, Sabin-like poliovirus type 1; SLPV-2, Sabin-like poliovirus type 2; SLPV-3, Sabin-like poliovirus type 3; NPEV, Non-polio Enterovirus; VDPV2, Vaccine derived poliovirus type 2, VDPV1; Vaccine Derived Poliovirus type 1, VDPV3; Vaccine Derived Poliovirus type 3

A total of 1266 sewage samples were collected from January 2020 to December 2021 from all 27 Egyptian governorates. One thousand seven hundred eighty-five (1785) positive isolates were detected from 1225 sewage samples and only 41 samples did not show viral growth (No CPE on both cell lines). In 2020, slightly more than one-third (36.68%) of the detected isolates were PVs of which 11.2% (*n* = 82) were SLPV1, 25.5% (*n* = 187) were SLPV3, (four isolates) one isolate (0.13%) for each of VDPV-1, VDPV-2, and two isolates (0.26%) of VDPV-3 and 461 isolates (63%) were Non-Polio Enteroviruses (NPEV) (Fig. 1a). However, in 2021, more than half the detected viruses were SLPVs divided into 8% (*n* = 85) SLPV-1; 18.6% (*n* = 196) SLPV-2; and 19.6% (*n* = 206) SLPV-3; 7.5% (*n* = 79) was VDPV2, 0.09% (*n* = 1) VDPV1 with absence of VDPV3 (Fig. 1b).

### Detection of polioviruses in stool samples

**Fig. 2:**
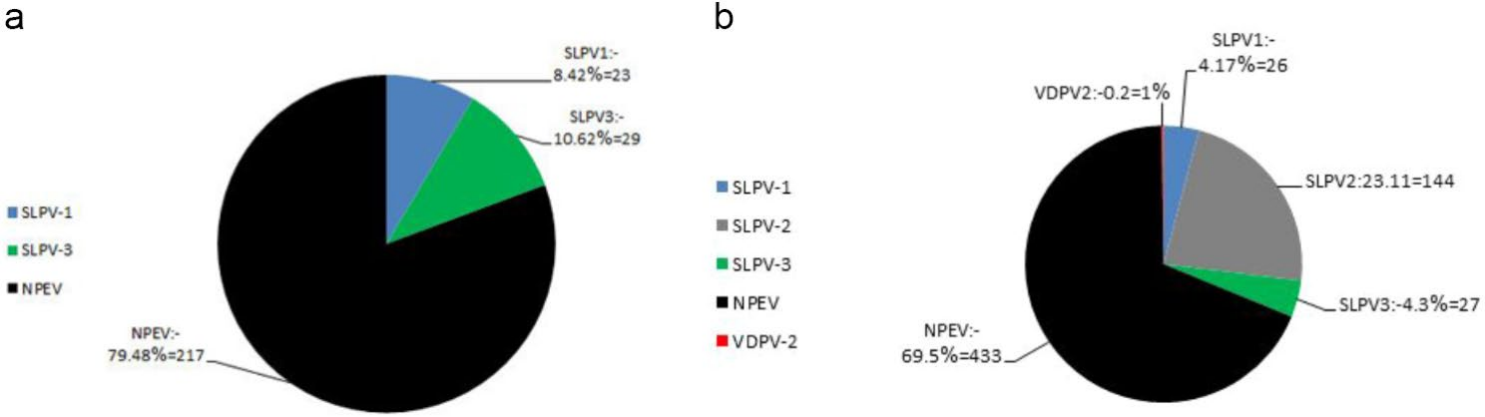
Distribution of detected viral Isolates from stool samples in the years 2020 (**a**) and 2021 (**b**). SLPV-1, Sabin-like poliovirus type 1; SLPV-2, Sabin-like poliovirus type 2; SLPV-3, Sabin-like poliovirus type 3; NPEV, Non-polio Enterovirus; VDPV2, Vaccine derived poliovirus type 2, VDPV1; Vaccine Derived Poliovirus type 1, VDPV3; Vaccine Derived Poliovirus type 3

From January 2020 to December 2021, a total of 4,357 stool specimens were collected from 2183 AFP cases across all 27 Egyptian governorates with (two samples collected 24 h apart for each case with few exceptions). Additionally, 1,336 stool specimens were collected from 1,336 contacts of these cases, with one sample taken from each contact. A total of 892 positive isolates varying between Polioviruses and NPEVs. In 2020, 269 isolates were obtained from inoculation with the following results: 8.42% (*n* = 23) SLPV-1, 10.62% (*n* = 29) SLPV-3 and 79.48% (*n* = 217) were NPEV (Fig. 2a). Moreover, in 2021, 623 isolates were obtained with the following results: 4.17% (*n* = 26) SLPV-1, 23.11% (*n* = 144) SLPV-2, 4.3% (*n* = 27) SLPV-3, 69.5%=433 NPEV and 0.2% (*n* = 1) VDPV2 (Fig. 2b).

### Monthly analysis

**Fig. 3:**
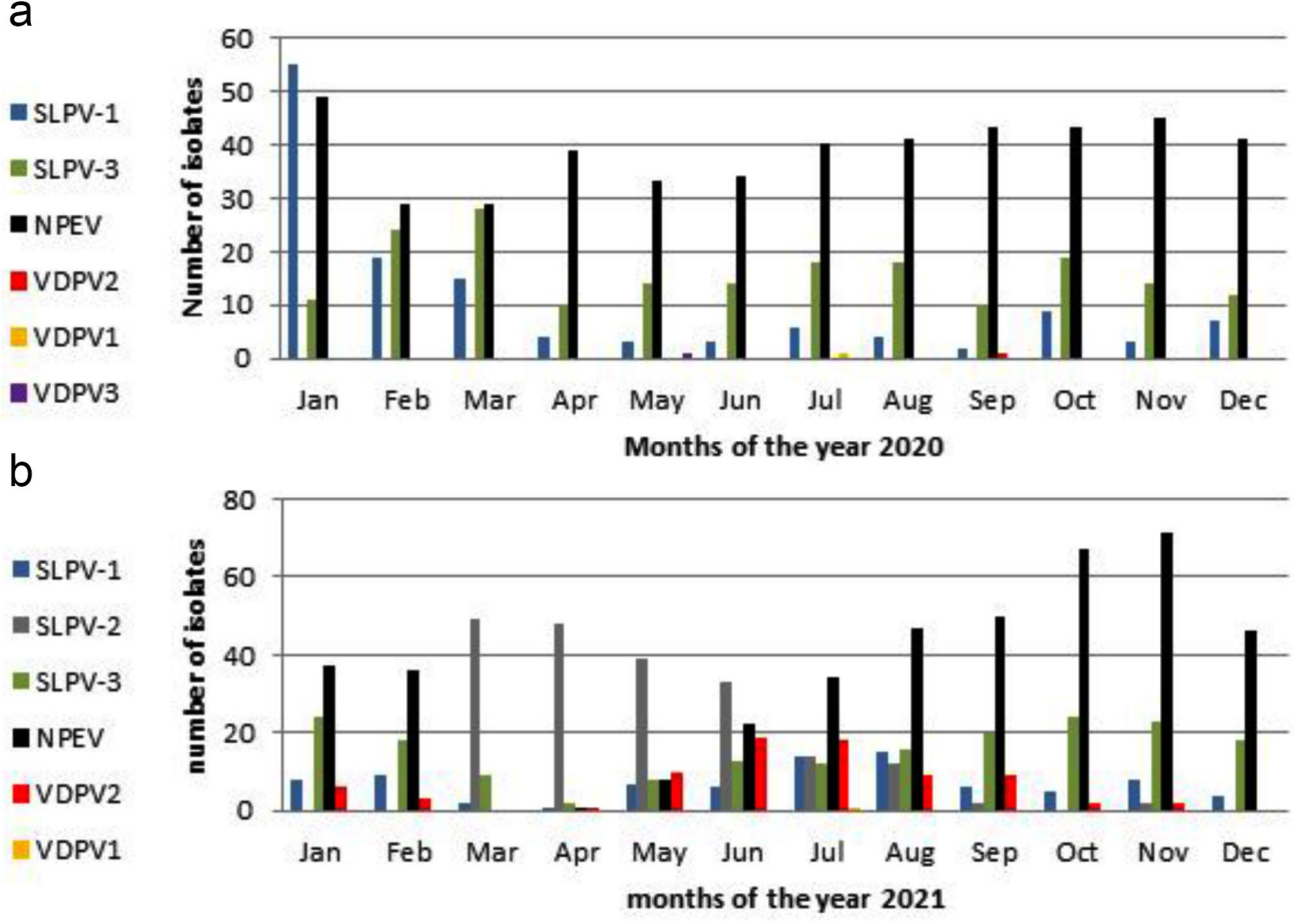
Distribution of detected viral Isolates from sewage samples on months of year 2020 (**a**) and 2021 (**b**). SLPV-1, Sabin-like poliovirus type 1; SLPV-2, Sabin-like poliovirus type 2; SLPV-3, Sabin-like poliovirus type 3; NPEV, Non-polio Enterovirus; VDPV2, Vaccine derived poliovirus type 2, VDPV1; Vaccine Derived Poliovirus type 1, VDPV3; Vaccine Derived Poliovirus type 3

For sewage samples, breaking up the recovered data by month in 2020 showed that both SLPVs and NPEVs were detected from sewage samples all year long, but their peak was in January (Fig. 3a). For clinical specimens, SLPVs appeared all year in small numbers, while NPEVs reached their peak during January, and September till November (Fig. 3a). In 2021, SLPVs isolates appeared in sewage samples throughout the year with a peak in March and April (Fig. 3b) in addition to NPEVs that recorded the highest number in November.

**Fig. 4:**
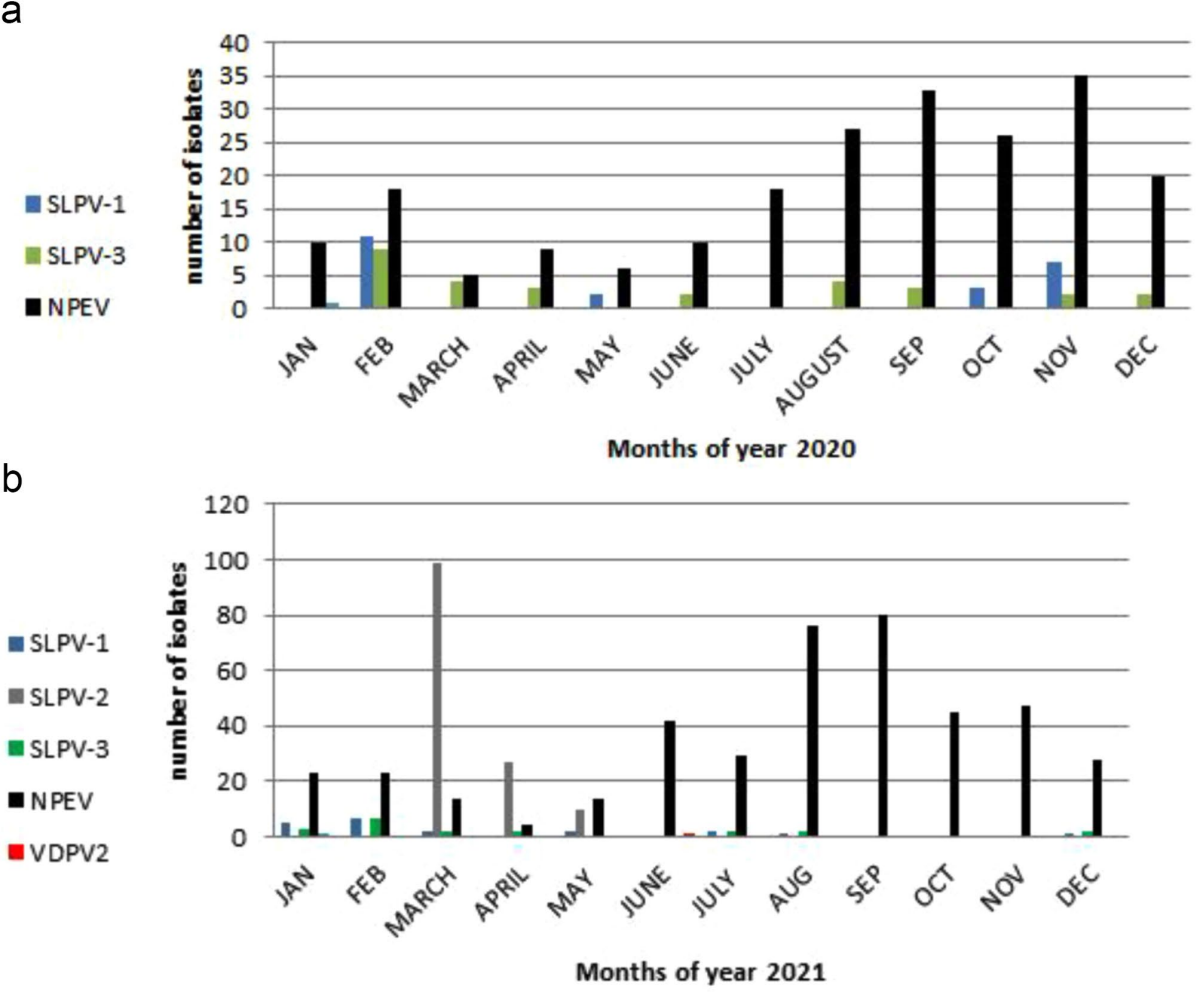
Distribution of detected viral Isolates from stool samples on months of year 2020 (**a**) and 2021 (**b**). SLPV-1, Sabin-like poliovirus type 1; SLPV-2, Sabin-like poliovirus type 2; SLPV-3, Sabin-like poliovirus type 3; NPEV, Non-polio Enterovirus; VDPV2, Vaccine derived poliovirus type 2, VDPV1; Vaccine Derived Poliovirus type 1, VDPV3; Vaccine Derived Poliovirus type 3

For stool samples, in 2020, SLPV-1 and SLPV-3 appeared mostly throughout the year with a peak in February (Fig. 4a) while November was the peak month for NPEVs. In 2021, March and April were also the peak months of SLPVs in stool samples, where most of them were SLPV-2 as shown in Fig. 4b, while September was the peak month for NPEVs.

### Analysis by governorate

**Fig. 5:**
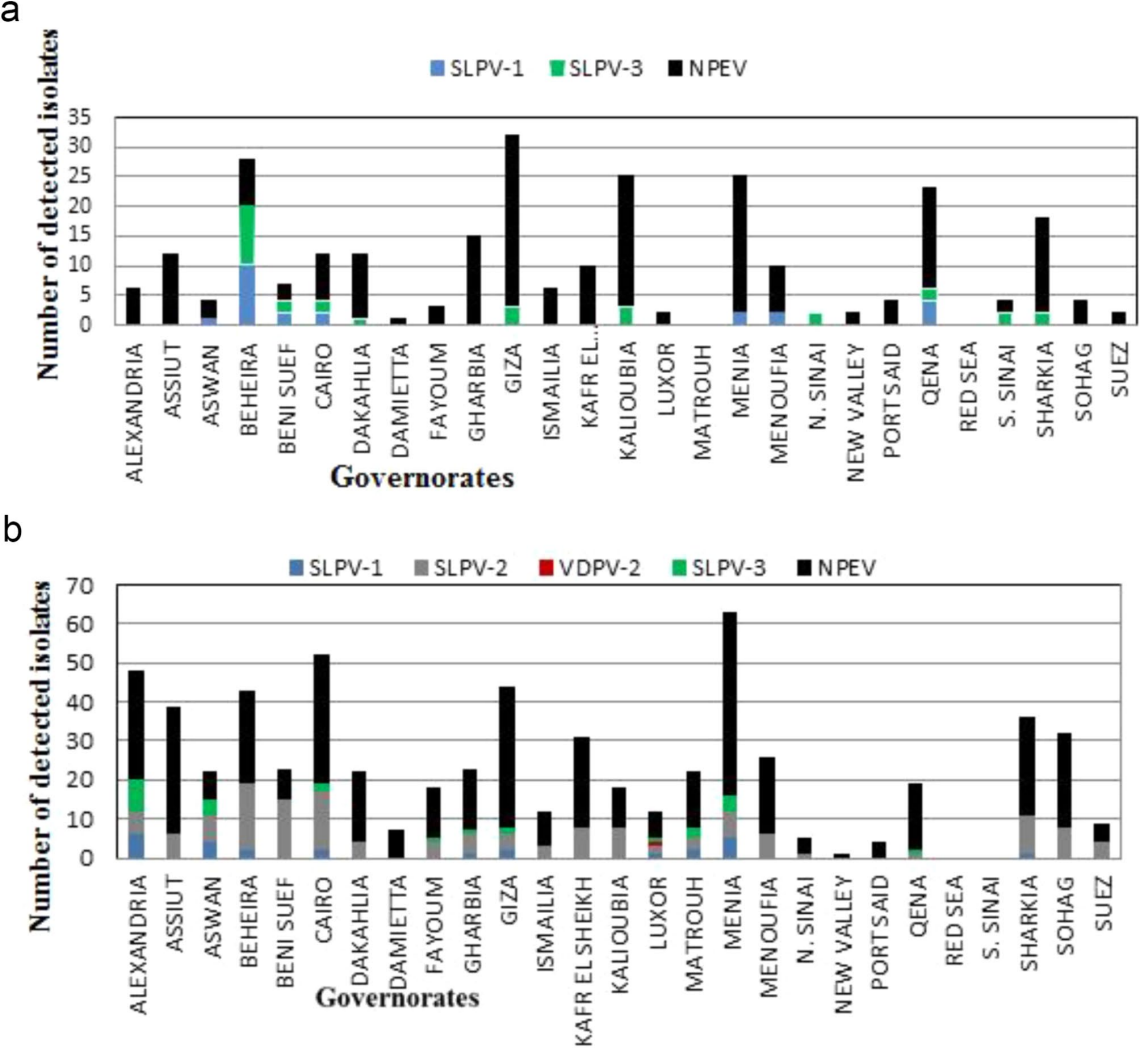
Distribution of detected viral Isolates from stool samples in different governorates of year 2020 (**a**) and 2021 (**b**). SLPV-1, Sabin-like poliovirus type 1; SLPV-2, Sabin-like poliovirus type 2; SLPV-3, Sabin-like poliovirus type 3; NPEV, Non-polio Enterovirus; VDPV2, Vaccine derived poliovirus type 2, VDPV1; Vaccine Derived Poliovirus type 1, VDPV3; Vaccine Derived Poliovirus type 3

For stool samples, in 2020, SLPVs (SLPV-1 and SLPV-3) in addition to NPEVs were detected in the 27 Egyptian governorates (Fig. 5a). As displayed in Fig. 5a, SLPVs were most detected in stool samples from the governate of Beheira, while NPEVs were most detected from the governate of Giza followed by Minya and Kalioubia. In 2021, the governorate of Minya reported the highest incidence of NPEV, with Giza following closely, while Cairo reported the highest incidence of SLPV most of which was SLPV-2 (Fig. 5b) as characterized by genetic sequencing. In addition, one of VDPV-2 was reported from the governate of Luxor (Fig. 5b).

**Fig. 6:**
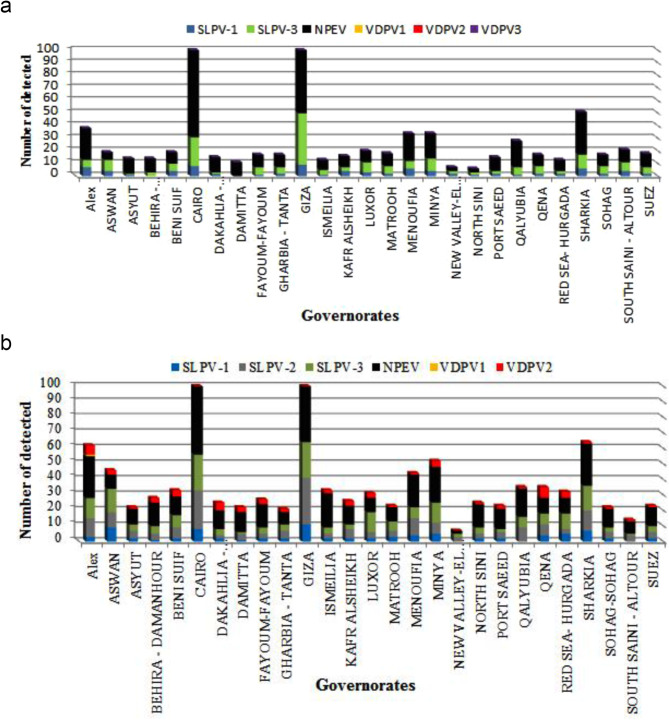
Distribution of detected viral Isolates from sewage samples in different governorates of year 2020 (**a**) and 2021 (**b**). SLPV-1, Sabin-like poliovirus type 1; SLPV-2, Sabin-like poliovirus type 2; SLPV-3, Sabin-like poliovirus type 3; NPEV, Non-polio Enterovirus; VDPV2, Vaccine derived poliovirus type 2, VDPV1; Vaccine Derived Poliovirus type 1, VDPV3; Vaccine Derived Poliovirus type 3

For sewage samples, in 2020 and as depicted in Fig. 6a, both SLPV and NPEV were most detected in sewage samples collected from Cairo and Giza with the latter recording the highest number of SLPV and were mostly SLPV-3. However, in 2021, the governates of Cairo and Giza reported the highest incidence of both SLPV (mostly SLPV-2) and NPEV followed by the governates of Sharkia and Alexandria (Fig. 6b). However, VDPV-2 was detected in 26 out of the 27 Egyptian governorates exhibiting a status of outbreak that required a proper and intervention (Fig. 6b).

### Detection of VDPVs during year 2020 and 2021

In the year 2020, four VDPVs were isolated: two of them were VDPV-3, one was VDPV-1 and one was VDPV-2 (Fig. 1a). It is worth noting that all 4 VDPVs were isolated from sewage samples collected from Giza governorate; one in May (VDPV-3), one in July (VDPV-1) and two during the month of September (VDPV-2 and VDPV-3). Besides, the VDPV positive samples also contained SLPV with or without NPEV (Table 1).

**Table 1:** Details of VDPVs isolated from sewage during the year 2020

Type	Governorate	Month	Sample	Number of nucleotides difference	%divergence	Other viruses detected in the sample
VDPV 1	Giza	July	Sewage	10	1%	SLPV-1 + NPEV
VDPV 2	Giza	September	Sewage	19	2%	SLPV-3 + NPEV
VDPV 3	Giza	May	Sewage	11	1%	SLPV-3
VDPV 3	Giza	September	Sewage	11	1%	SLPV-3 + NPEV

VDPVs, Vaccine Derived Poliovirus; NPEV, Non-polio Enteroviruses; SLPV, Sabin-like Poliovirus

In the year 2021, the VDPV-2 isolated from sewage samples were 79 isolates from different sites & governorates, of which six isolates were detected in January then three in February from six governorates in Egypt. Worthwhile, these nine isolates have shown substantial genetic divergence from Sabin 2 reference strain in the VP1 region and were classified as cVDPV2 confirming a possible start of an outbreak caused by circulating vaccine-derived poliovirus type 2.

As a response to these implications, the Expanded Program of Immunization (EPI) in MOHP launched two consecutive vaccination campaigns by monovalent type 2 OPV through year 2021. The first started at the end of February followed by a second one at the end of March 2021. Despite of the above response, new 70 VDPV-2 isolates continued to be detected; based on sequence analysis, only 2 of them were classified as cVDPV2 (one was detected in June and the second in November 2021) while the other 68 were found to be not genetically linked to any previously isolated VDPV2 and were later classified as ambiguous VDPV2s (aVDPV-2). In addition to the above VDPV2s, only one VDPV1 was detected in a sewage sample collected from Alexandria in July which showed 19 nucleotide changes (about 2% divergence) from reference Sabin1 strain & was later classified as aVDPV1. The origin, month of detection, % nucleotide divergence and the NCBI GenBank accession codes for all VP1 sequences of the 11 cVDPV2s & 68 aVDPV2s isolated from sewage in 2021 are shown in Tables 2 and 3, respectively.

**Table 2:** Details of circulating vaccine derived poliovirus (cVDPVs) isolated from sewage samples during the year 2021

Nr	Type of PV	Governorate	Month	Number of nucleotides differences from Sabin 2	%divergence	Other viruses detected in the sample	NCBI accession codes
1	cVDPV-2	Alexandria	January	20	2%	NPEV	OR345204
2	cVDPV-2	Aswan	January	23	2%	SLPV-1 + SLPV-3 + NPEV	OR345205
3	cVDPV-2	Qena	January	20	2%	NPEV	OR345207
4	cVDPV-2	Banisuif	January	20	2%		OR345208
5	cVDPV-2	Kafr Elsheikh	January	20	2%	NPEV	OR345209
6	cVDPV-2	Giza	January	20	2%	SLPV-3 + NPEV	OR345210
7	cVDPV-2	Aswan	February	22	2%	SLPV-3	OR345206
8	cVDPV-2	Qena	February	19	2%	SLPV-1 + SLPV-3	OR345211
9	cVDPV-2	Alexandria	February	21	2%	SLPV-3 + NPEV	OR345212
10	cVDPV-2	Qena	June	23	2%	SLPV-1	OR365429
11	cVDPV-2	Giza	November	15	1%	SLPV-1 + NPEV	OR260447

cVDPVs, circulating Vaccine Derived Poliovirus; NCBI, National Center for Biotechnology Information; NPEV, Non-polio Enteroviruses; SLPV, Sabin-like Poliovirus

**Table 3:** Details of ambiguous vaccine derived poliovirus (aVDPVs) isolated from sewage samples during the year 2021

Nr.	Type	Governorate	Month	Number of different nucleotides	%divergence	Other viruses detected in the sample	NCBI accession codes
1	VDPV-2	Cairo	April	8	1%	-	OR345213
2	VDPV-2	Giza	May	8	1%	-	OR345214
3	VDPV-2	Menoufia	May	6	1%	-	OR345215
4	VDPV-2	Aswan	May	6	1%	SLPV-3	OR365418
5	VDPV-2	Port saeed	May	7	1%	SLPV-3	OR365420
6	VDPV-2	Giza	May	8	1%	-	OR365421
7	VDPV-2	Dakahlia	May	10	1%	-	OR365422
8	VDPV-2	Luxor	May	6	1%	SLPV-3	OR365423
9	VDPV-2	Alexandria	May	7	1%	-	OR365424
10	VDPV-2	Giza	May	7	1%	-	OR365425
11	VDPV-2	Qena	May	7	1%	-	OR365419
12	VDPV-2	Domietta	June	6	1%	-	OR365426
13	VDPV-2	Giza	June	6	1%	NPEV	OR365427
14	VDPV-2	Cairo	June	7	1%	NPEV	OR365428
15	VDPV-2	Suez	June	7	1%	SLPV-3 + NPEV	OR365430
16	VDPV-2	Minya	June	7	1%	-	OR365431
17	VDPV-2	Alexandria	June	9	1%	NPEV	OR365432
18	VDPV-2	Kafr alsheikh	June	7	1%	-	OR365433
19	VDPV-2	Beni suif	June	7	1%	NPEV	OR365434
20	VDPV-2	Fayoum	June	7	1%	NPEV	OR365435
21	VDPV-2	Giza	June	8	1%	SLPV-3	OR365436
22	VDPV-2	Sharkia	June	6	1%	SLPV-3 + NPEV	OR365437
23	VDPV-2	Red sea	June	8	1%	SLPV-3	OR388075
24	VDPV-2	Luxor	June	6	1%	SLPV-3	OR388076
25	VDPV-2	Dakahlia	June	9	1%	NPEV	OR388077
26	VDPV-2	Alexandria	June	7	1%	-	OR388078
27	VDPV-2	Beni suif	June	8	1%	NPEV	OR388079
28	VDPV-2	Giza	June	6	1%	NPEV	OR345203
29	VDPV-2	North sinia	June	8	1%	NPEV	OR388080
30	VDPV-2	Domietta	July	6	1%		OR388081
31	VDPV-2	Behira	July	9	1%	SLPV-3 + NPEV	OR388082
32	VDPV-2	Fayoum	July	11	1%	SLPV-3 + NPEV	OR388083
33	VDPV-2	Gharbia	July	8	1%	SLPV-1	OR388084
34	VDPV-2	Sohag	July	6	1%	-	OR428200
35	VDPV-1	Alexandria	July	19	2%	SLPV-3	-
36	VDPV-2	Alexandria	July	7	1%	SLPV-1 + NPEV	OR428201
37	VDPV-2	Menya	July	8	1%	SLPV-1 + NPEV	OR428202
38	VDPV-2	Kafr alsheikh	July	8	1%	NPEV	OR428203
39	VDPV-2	Giza	July	7	1%	-	OR428204
40	VDPV-2	Fayoum	July	6	1%	NPEV	OR428205
42	VDPV-2	Matrooh	July	7	1%	NPEV	OR428206
43	VDPV-2	Dakahlia	July	7	1%	NPEV	OR428207
44	VDPV-2	Ismaeilia	July	8	1%	SLPV-1 + SLPV-3 + NPEV	OR428208
45	VDPV-2	Qena	July	6	1%	SLPV-1	OR428209
46	VDPV-2	Kafr alsheikh	July	8	1%	NPEV	OR428210
47	VDPV-2	Behira	July	8	1%	SLPV-3 + NPEV	OR428211
48	VDPV-2	Cairo	July	7	1%	NPEV	OR428212
49	VDPV-2	Port saeed	July	7	1%	SLPV-1 + NPEV	OR428213
50	VDPV-2	Domietta	August	11	1%	NPEV	OR2732608
51	VDPV-2	Minya	August	6	1%	NPEV	OR478894
52	VDPV-2	Dakahlia	August	6	1%	SLPV-3	OR478895
53	VDPV-2	Luxor	August	7	1%	SLPV-3	OR478896
54	VDPV-2	Qalubia	August	11	1%	-	OR478897
55	VDPV-2	Cairo	August	10	1%	-	OR478898
56	VDPV-2	Red sea	August	11	1%	SLPV-1	OR478899
57	VDPV-2	Giza	August	9	1%	NPEV	OR478900
58	VDPV-2	Behira	August	10	1%	SLPV-3 + NPEV	OR478901
59	VDPV-2	Minya	September	10	1%	SLPV-3 + NPEV	OR597540
60	VDPV-2	Giza	September	7	1%	SLPV-3 + NPEV	OR597541
61	VDPV-2	Red sea	September	6	1%	SLPV-3 + NPEV	OR597542
62	VDPV-2	Gharbia	September	13	1%	NPEV	OR597543
63	VDPV-2	Qena	September	13	1%	NPEV	OR597544
64	VDPV-2	South saini	September	6	1%	-	OR597545
65	VDPV-2	Dakahlia	September	8	1%	NPEV	OR597546
66	VDPV-2	Red sea	September	10	1%	SLPV-1 + SLPV-3 + NPEV	OR597547
67	VDPV-2	Qena	September	10	1%	SLPV-1 + SLPV-3	OR597548
68	VDPV-2	Beni suif	October	8	1%	SLPV-3 + NPEV	OR597549
69	VDPV-2	Cairo	November	17	2%	NPEV	OR597551

aVDPVs, Ambiguous Vaccine Derived Poliovirus; NCBI, National Center for Biotechnology Information; NPEV, Non-polio Enteroviruses; SLPV, Sabin-like Poliovirus

On the other hand, in 2021 only one stool sample was VDPV-2, it was collected from a contact from LUXOR in June 2021 and upon sequencing it showed 7 nucleotide differences (about 0.78%) divergence in VP1 region from reference Sabin 2 strain was later classified as aVDPV2. The nucleotide sequence of the VP1 region of this isolate was also submitted into the NCBI GenBank under the accession code, PP481414.

### Molecular epidemiology of detected VDPV2

**Fig. 7:**
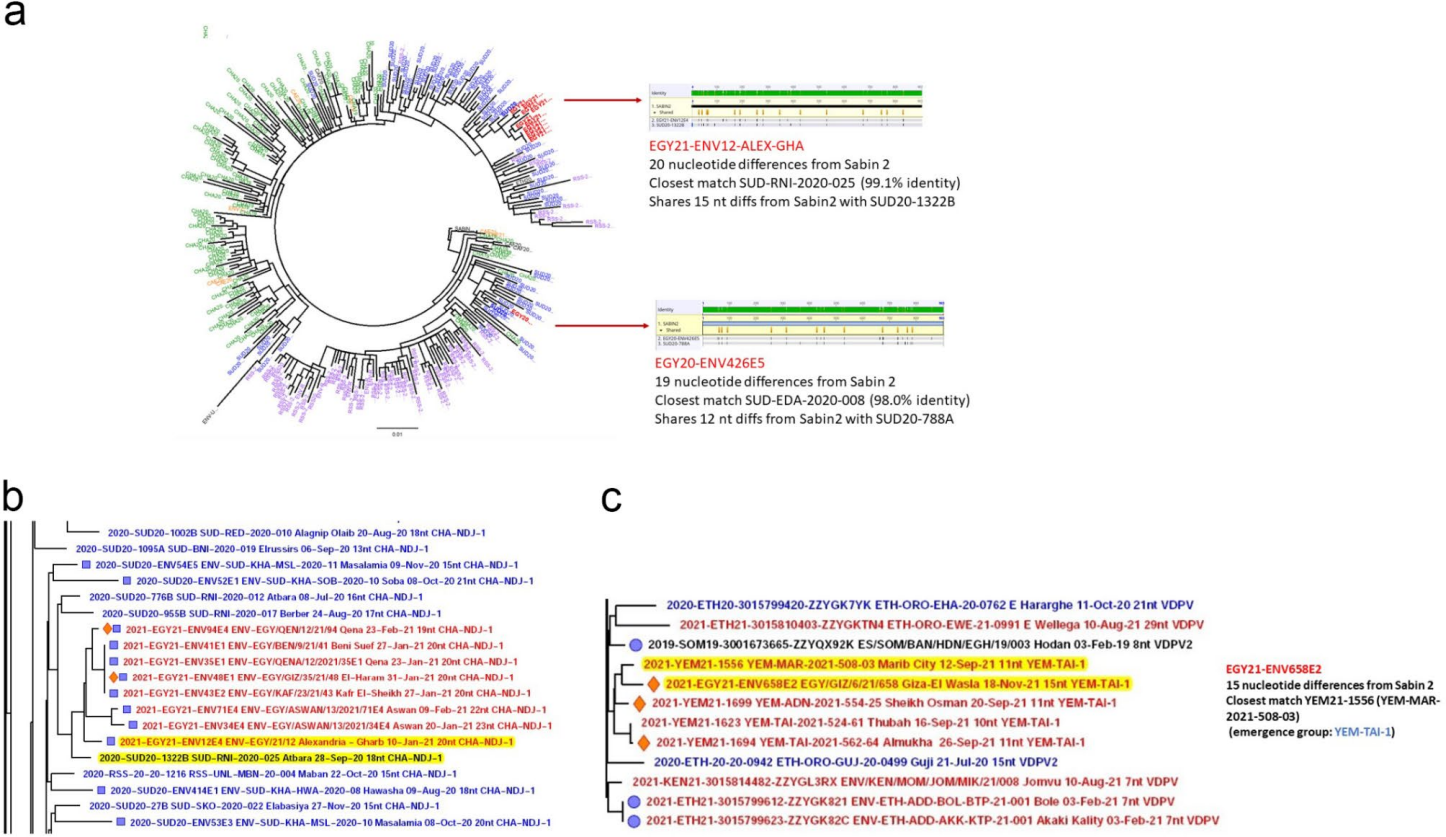
Phylogenetic analysis of VDPV-2 obtained in 2021 and Emergency groups (**a**) Phylogenetic analysis of Alex sample and Sudan emergencu group (**b**) cVDPVs and their relativity to emergency group SUD (**b**) Giza samples and relativity to YEM emergency group

Phylogenetic analysis of the isolated VDPV2s has revealed that the first VDPV2 sequence isolated from sewage water collected in September 2020 from Giza (Site: Al-Eshreen) had 19 nucleotide differences from Sabin2 in VP1 gene and was genetically linked to a VDPV2 previously isolated from a stool sample from Sudan (IDcode: SUD-EDA-2020-008, emergence group: CHA-NDJ-1). No other VDPV2s were detected for the next 3 months until January 2021 where sewage sample EGY21-ENV12-ALEX (collected from Alexandria, site: Gharb)) was found to have a VDPV2 with 20 nucleotide differences in its VP1 gene from reference Sabin2 and was genetically linked to the cVDPV2 sequence from another stool sample from Sudan (IDCode: SUD-RNI-2020-025, Atbara district, emergence group CHA-NDJ-1) as shown in Fig. 7a. Shortly in January, another VDPV2 sequence was detected in a sewage sample from Aswan (IDCode: *EGY21-ENV34-ASW)* which had 23 nucleotide differences from reference Sabin2 in VP1, and was genetically linked to the cVDPV-2 sequence of sample EGY21-ENV12-ALEX-GHA. Eight of the remaining 9 cVDPV2s detected in sewage samples of 2021 were all linked to the same Egyptian sewage sample EGY21-ENV12-ALEX from Alexandria and hence were related to the same emergence group CHA-NDJ-1 with nucleotide changes in their VP1 ranging from 19 to 23 nucleotides (Fig. 7b). The last cVDPV2 in 2021 was detected in November in a sewage sample collected from Giza (IDCode: *EGY21-ENV658-GIZ)* and had 15 nucleotide differences from Sabin 2, but was found to be genetically linked to a cVDPV2 isolated from a stool sample from Yemen (IDCode: YEM-MAR-2021-508-03, Maarib City district) belonging to a different emergence group YEM-TAI-1 (Fig. 7c). Apart from the above mentioned cVDPV2s, a total of 68 VDPV2 sequences were detected without any genetic linkages to previously isolated VDPV2s and hence, were later classified as aVDPV2 with 6–19 nucleotide changes in their VP1.

Statistical analysis revealed that the nucleotide differences in all detected type 2 Polioviruses were in 252 positions by different number of repeats, position number *428* being the most common in VP1 region, was repeated 53 times with (*p* = 0.093), followed by 13 mutations in position number 13 with (*p* = 0.023).

## Discussion

The Global Polio Eradication Initiative (GPEI) is now at one of its most crucial turn points and errors will be extremely costly [14, 15]. Thus, continuous and careful monitoring of stool specimens from AFP cases and sewage samples is a key player for the polio endgame [14]. Egypt was added by the WHO to the list of polio-free countries in 2006 and the final phase of eradication was commenced in Egypt in 2018 [6]. Thus, the presence of a robust national polio surveillance system is imperative to make sure no new cases are imported from outside the country, or variants arise from vaccine strains [16]. As per the Polio Eradication Strategy 2022–2026, outbreaks of cVDPV2 represent a global concern in the current stage of polio eradication. One of the two main goals upon which this strategy was built, is to stop cVDPV transmission and prevent outbreaks in non-endemic countries. Therefore, maintaining a sensitive surveillance system to quickly detect and rapidly respond to any cVDPV outbreak is a must for achieving this goal [17].

Like most infectious agents, polioviruses can overcome borders, putting unimmunized individuals anywhere at risk. As shown in the above results, genetic linkages found between the cVDPV2s identified in wastewater in Egypt and other VDPV2s isolated from AFP cases in Sudan, Chad and Yemen provide a clear indication of this risk. VDPVs are actually a true threat to polio eradication, being a key factor in the reoccurrence of paralytic polio hand in hand with vaccine refusal [13]. Based on previous studies in other countries, the VDPVs have caused epidemics in above 25 countries in Africa, the Middle East, South eastern Asia and Pacific west between Jan. 2018 and June 2019 [11]. For example, in 2019 only, 350 or more cVDPD2-induced AFP cases were recorded in 13 countries in Africa [14].

In this study we report the findings of routine poliovirus surveillance in Egypt prior to and during the early stage of a VDPV2 outbreak. Results have shown that during the year 2020, nearly all polioviruses isolated from both stool and sewage samples were of the Sabin-like genotype (with both PV1 & PV3 serotypes) except for 4 isolates that showed vaccine-derived polioviruses, two of which were VDPV-3, one was VDPV-1 and one was VDPV-2, all from sewage samples from Giza governorate. Both VDPV1 & VDPV3 sequences were not genetically linked to any previously detected VDPV while the VDPV2 sequence was genetically linked to a cVDPV2 from a Sudanese isolate belonging to CHA-NDJ-1 emergence group and hence, was considered as an importation event of a cVDPV2 in Egypt. Continuous monitoring of sewage samples for the next 3 months showed no further detection of any VDPV2 neither in Giza nor in all other governorates and so was the case with stool samples. In January 2021, a second VDPV2 was detected in sewage from Alexandria and was found to be genetically linked to a cVDPV2 from another Sudanese isolate belonging also to CHA-NDJ-1 emergence group. This was shortly, in the same month, followed by the detection of five more VDPV2s in five different governorates which were found to be genetically linked to Alexandria VDPV2 & hence, were immediately classified as cVDPV2 indicating a community circulation of this cVDPV2 inside Egypt & confirming a start of a cVDPV2 outbreak. Three more cVDPV2s were detected in February from subsequent monthly samples before the launch of the first massive mOPV2 vaccination campaign at the end of February initiated by the Egyptian health authorities in coordination with & support from the WHO in an attempt to minimize the chance of VDPV2 transmission and to stop its circulation. The second massive vaccination campaign by mOPV2 was at the end of March, where the laboratory results for the period between the two campaigns showed no further detection of cVDPV2 with only SLPV2 being detected in both sewage and stool samples which is a normal observation after vaccination campaigns by OPV. Starting from April 2021, more VDPV2s began to appear in sewage samples side by side with the SLPV2. VP1 sequences of these VDPV2s showed a fewer number of nucleotide changes with no genetic linkage to previously isolated VDPV2s indicating a secondary seeding of VDPV2 caused by the mOPV2 used in vaccination campaigns. The last cVDPV2 belonging to CHA-NDJ-1 emergence group was detected in June in a sewage sample from Qena with no further detection of any cVDPV2 to the end of the year except for a single importation event of a cVDPV2 detected in Giza that was found to be related to another emergence group originated in Yemen (YEM-TAI-1). On the other hand, ambiguous VDPV2s continued to be detected in sewage to the end of the year with only one incidence in a stool sample from Luxor. The forementioned findings indicate that the use of mOPV2 in the 2 vaccination campaigns successfully stopped the circulation of the CHA-NDJ-1 cVDPV2, but in the same time led to the seeding of new VDPV2s in immunized children which might be due to insufficient vaccination coverage. The above-mentioned observation clearly aligns with the strategic objectives and key activities of the second goal of the Polio Eradication Strategy 2022–2026 which highlight the importance of improving campaign planning & execution to control cVDPV outbreaks but in the same time direct toward the importance of using genetically stable oral polio vaccines, like nOPV2, to minimize the risk of outbreak seeding [17].

It is worth noting that nearly all detected VDPV2, both circulating (*n* = 11) and ambiguous (*n* = 68) were isolated from sewage with only one VDPV2 isolated from a stool sample collected from a contact to an AFP case, an observation that strongly draws the attention to the critical role of environmental surveillance, especially, in this stage of polio eradication.

Sewage can be used as a holistic approach to study the epidemiology and genetic divergence of enteroviruses present, including polioviruses. In the case of our study, environmental surveillance through the investigation of sewage samples was capable of detecting many cVDPV2s several months before obtaining a single detection in only one stool sample.

In this study, we checked for viruses in sewage samples (*n* = 1266) from all Egyptian governorates through the COVID-19 years, 2020 and 2021. Our results have shown that 94.7% of isolates contained one or more viruses. High levels of viruses are common in developing countries [18, 19]. The source of these viruses is stool from infected cases and/or carriers, as well as improper disposal of hospital and laboratory waste [20]. Furthermore, our study highlights the crucial role of timely investigation and response to VDPV detections, which involves rapidly identifying and containing outbreaks through targeted vaccination campaigns and intensified environmental surveillance. These measures are essential for preventing further transmission of the virus and restricting its geographical spread [21, 22].

Results have shown that the number of isolated viruses from sewage samples collected from Giza governorate in 2020 and 2021 was by far the highest, whether those viruses were Polio (SLPV or VDPV) or NPEVs, second comes Cairo, the capital governorate. This could be explained by the fact that these are the two most heavily populated governorates hosting about 9.2 and 10.3 million people, respectively [23], in addition to being target governorates for foreign populations moving from neighboring countries.

Studying poliovirus in sewage is a great tool to take an overview for the health status of a specific area [24]. The large number of circulating vaccine derived poliovirus is evidence of person-to-person transmission. MOHP and GPEI have played a crucial role in the outbreak response, and to avoid the aggravation of the situation, launching of two monovalent OPV consecutive campaigns were a necessity to stop further transmission [24]. Genetic analysis of poliovirus helps in tracing transmission chains and understanding how the virus spreads within a region or between countries. Additionally, efforts should be directed towards developing safer and more effective polio vaccines that have a reduced likelihood of generating VDPVs.

It was previously reported that delayed and improper Lab analysis and low immunization of children against polio were the main cause of cVDPV1 outbreak in Yemen 2020 [25] and cVDPV2 outbreak in Yemen 2021–2022 [26]. However, in Egypt, after the successful monitoring of VDPV2 by the national polio surveillance system as well as the quick response to such cVDPV2 outbreak by the Egyptian (MOHP) in collaboration with WHO, no polio cases have been reported in the country during the period of this study. However, the aVDPV2s caused by secondary seeding continued to be detected, which prompted the national health authorities, upon recommendation from the WHO, to start the deployment of nOPV2 in vaccination at the end of year 2021.

## Conclusion

This study highlights the importance of having a strong and vigilant national polio surveillance system with a special focus on the crucial role of environmental surveillance in the early detection and combating of outbreaks caused by vaccine-derived polioviruses. Results and findings of the current study also demonstrated that early and prompt response to the VDPV2 outbreaks by boosting immunization through the use of mOPV2 in vaccination campaign is capable of controlling the outbreak and stopping the further transmission of cVDPV2 but with paying special attention to the possibility of secondary seeding of new VDPV2s if the vaccination coverage was compromised. This serious drawback of mOPV2 should draw attention to the importance of developing and using more genetically stable OPVs, e.g. nOPV2, which in the same time should be of comparable efficiency to the ordinary mOPV2.

### Electronic supplementary material

Below is the link to the electronic supplementary material.

Supplementary Material 1

## Data Availability

All data generated or analyzed during this study are included in this published article in the main manuscript and supplementary file. The nucleotide sequences of all the detected vaccine-derived poliovirus (VDPVs) included in this study VDPV have been annotated and deposited in the NCBI GenBank database https://www.ncbi.nlm.nih.gov/ and are publicly available under the respective accession codes that are provided in the manuscript, Tables 2, and 3.

## Abbreviations

AFP: Acute flaccid paralysis
aVDPV: Ambiguous vaccine derived poliovirus
cVDPV: Circulating vaccine derived poliovirus
CDC: Center of Disease control
ES: Environmental samples
EPI: Extended Program of Immunization
GPEI: Global Polio Eradication Initiative
mOPV: Monovalent oral Polio vaccine
NPEV: Non-Polio Enterovirus
OPV: Oral Poliovirus vaccine
SLPV: Sabin-like Poliovirus
VDPV: Vaccine Derived Poliovirus
SIA: Supplementary Immunization Activity
