# Supplementary material for: Monitoring the VDPV2 outbreak in Egypt during 2020–2021 highlights the crucial role of environmental surveillance and boosting immunization in combating Poliovirus
Source: BMC Infect Dis. 2024 Aug 26;24:866. doi: 10.1186/s12879-024-09731-0 (PMC11348703; doi:10.1186/s12879-024-09731-0)
Supplement: Supplementary file 1 — Supplementary Material 1 [file 12879_2024_9731_MOESM1_ESM.docx]

**Table S1.** List of targets, primers and oligonucleotide Sequence of primers used in this study

| **Target** | **Primer** | **Oligonucleotide sequence (5’-3’)** | **Position** |
| --- | --- | --- | --- |
| Sabin 1 | Sabin 1 2S | AGG TCA GAT GCT TGA AAG C | 2505–2523 |
|  | Sabin 1 1A | CCA CTG GCT TCA GTG TTT | 2600–2583 |
| Sabin 2 | Sabin 2 2S | CCG TTG AAG GGA TTA CTA AA | 2525–2544 |
|  | Sabin 2 1A | CGG CTT TGT GTC AGG CA | 2595–2579 |
| Sabin 3 | Sabin 3 2S | AGG GCG CCC TAA CTT T | 2537–2552 |
|  | Sabin 3 1A | TTA GTA TCA GGT AAG CTA TC | 2591–2572 |
| PanEV (any EV) | PAN-EV S | GGC CCC TGA ATG CGG CTA ATC C | 458–480 |
|  | PAN-EV A | GCG ATT GTC ACC ATW AGC AGY CA | 603–581 |
| PanPV (any poliovirus) | PanPV/PCR-S1 | TTG GAG TTC TTC ACI TAI TCI MGI TTY GAY ATG | 2832–2864 |
|  | PanPV PCR-1A | GGA GCT CCG GGT GGG AYR TAC ATI ATY TGR TAI AC | 2962–2928 |
| WPV1 (wild PV1) | WEAF WPV1 S | GTA CAA ACC AGT CAY GTN AT | 2661–2680 |
|  | SOAS WPV1 S | CGT ACA GAC TAG RCA YGT NAT | 2660–2680 |
|  | WPV1 A | GAG AAT AAY TTG TCY TTK GAY GT | 2800–2778 |
| PV2 (any serotype 2) | PV Type 2 S | GAT GCA AAY AAC GGI CAT GC | 2911–2930 |
|  | PV Type 2 A | TCA TAA AAG TGG GAR TAC GCR TT | 3110–3088 |
| AFR WPV3 | | | |
| WPV3-I | SOAS WPV3 S | CAG GGA GTA GAT GAY CTN AT | 2443–2462 |
|  | WEAF WPV3 S | CAG GGG GTT GAT GAY TTR AT | 2443–2462 |
| WEAF genotype | WPV3 A | ACK GTG TCT GAY GGN AC | 2623–2607 |
| SOAS WPV3 | | | |
| WPV3-II | SOAS 6S | GTY RTA CAR CGR CGY AGY AGR A | 2671–2692 |

**Table S2.** The target gene and primer locations for each target virus used in this study

| Name | Gene | S/A | Reference virus | Base start | Base End | Sequence | Target virus |
| --- | --- | --- | --- | --- | --- | --- | --- |
| 246S-S1 | VP3 | S | Sabin1  Genbank  AY184219 | 2441 | 2460 | CGAGATACCACATATAGA | Sabin 1 |
| 249S-S1 | VP1 | A | Sabin1  Genbank  AY184219 | 2800 | 2782 | CACTGTAAATAGCTTATCC | Sabin 1 |
| 249S-S1 | VP1 | S | Sabin1  Genbank  AY184219 | 2782 | 2800 | GGATAAGCTATTTACAGTG | Sabin 1 |
| 252A-S1 | VP1 | A | Sabin1  Genbank  AY184219 | 3385 | 3364 | ATATGTGGTCAGATCCTTGGTG | Sabin 1 |
| 247S-S2 | VP3 | S | Sabin2  Genbank  AY184220 | 2443 | 2462 | CGAGATACAACACACATTAG | Sabin 2 |
| 250A-S2 | VP1 | A | Sabin2  Genbank  AY184220 | 2802 | 2784 | AACCGAAAACAATCTGCTG | Sabin 2 |
| 250S-S2 | VP1 | S | Sabin2  Genbank  AY184220 | 2784 | 2802 | CAGCAGATTGTTTTCGGTT | Sabin 2 |
| 253A-S2 | VP1 | A | Sabin2  Genbank  AY184220 | 3384 | 3363 | ATAAGTCGTTAATCCCTTTTCT | Sabin 2 |
| 248S-S3 | VP3 | S | Sabin 3  Genbank  AY184221 | 2438 | 2457 | CGAGACACCACTCACATTTC | Sabin 3 |
| 251A-S3 | VP1 | A | Sabin 3  Genbank  AY184221 | 2791 | 2773 | CATGGCAAATAGTTTCTGT | Sabin 3 |
| 251S-S3 | VP1 | S | Sabin 3  Genbank  AY184221 | 2773 | 2791 | ACAGAAACTATTTGCCATG | Sabin 3 |
| 254A-S3 | VP1 | A | Sabin 3  Genbank  AY184221 | 3377 | 3356 | ATATGTGGTTAATCCTTTCTCA | Sabin 3 |
